# Diagonal queue medical image steganography with Rabin cryptosystem

**DOI:** 10.1007/s40708-016-0032-8

**Published:** 2016-02-15

**Authors:** Mamta Jain, Saroj Kumar Lenka

**Affiliations:** Department of Information Technology, Mody University of Science and Technology, Lakshmangarh, Rajasthan India

**Keywords:** Diagonal queue, Brain disease image, Steganography, Cryptography, Encryption, Decryption, Embedding

## Abstract

The main purpose of this work is to provide a novel and efficient method to the image steganography area of research in the field of biomedical, so that the security can be given to the very precious and confidential sensitive data of the patient and at the same time with the implication of the highly reliable algorithms will explode the high security to the precious brain information from the intruders. The patient information such as patient medical records with personal identification information of patients can be stored in both storage and transmission. This paper describes a novel methodology for hiding medical records like HIV reports, baby girl fetus, and patient’s identity information inside their Brain disease medical image files viz. scan image or MRI image using the notion of obscurity with respect to a diagonal queue least significant bit substitution. Data structure queue plays a dynamic role in resource sharing between multiple communication parties and when secret medical data are transferred asynchronously (secret medical data not necessarily received at the same rate they were sent). Rabin cryptosystem is used for secret medical data writing, since it is computationally secure against a chosen-plaintext attack and shows the difficulty of integer factoring. The outcome of the cryptosystem is organized in various blocks and equally distributed sub-blocks. In steganography process, various Brain disease cover images are organized into various blocks of diagonal queues. The secret cipher blocks and sub-blocks are assigned dynamically to selected diagonal queues for embedding. The receiver gets four values of medical data plaintext corresponding to one ciphertext, so only authorized receiver can identify the correct medical data. Performance analysis was conducted using MSE, PSNR, maximum embedding capacity as well as by histogram analysis between various Brain disease stego and cover images.

## Introduction

The wide applications of various forms of networks and the Internet of the Things (IoT) as well as simultaneously the growing cloud computing architecture with the big data complexity are the remarkable challenge to the image steganography. In recent times, various government and private medical organizations are continuously migrating into the cloud and mobile environments with the increasing use of networking services. These systems require large-scale medical data centers for medical data storage. In store-forward version of the telemedicine, the doctor examines the medical image along with the patient data which are transmitted from remote places. Since the medical images and the data are transmitted from remote places, security parameters such as authentication, integrity, confidentiality, and availability have to be taken care of. The advent of high-speed Internet technology has changed the way patients receive medical care by expediting diagnosis and allowing immediate treatment. However, this incredible tool comes with a security onus, especially when sensitive patient information is transmitted. The Department of Health and Human Services (DHHS), in fact, imposed regulations for data security and privacy under the Health Insurance and Portability and Accountability Act (HIPAA) of 1996 [1, 2]. In recent years, several architectures for secure storage and transmission of medical records and patient identification information have been proposed, both for real- and semi-real-time applications like blood glucose monitoring, secure telemedicine [3], and non-real-time applications that involve maintaining and sharing medical records and databases. Most of these architectures, however, rely on some form of cryptography. Cryptographic techniques encrypt the medical records with a password and assume that only authorized parties have access to the password. While this does work most of the time, the encrypted data are prone to prying security thieves, who could decipher sensitive information like the patients’ insurance service provider, medication history, etc. Steganography provides an extremely effective alternative to this problem, hiding the very existence of sensitive data by concealing the data in a “carrier.” The objective of image steganography is to hide secret information in nondescript areas of the carrier image such that the changes made to the image are imperceptible, and the secret information itself is retrievable only by authorized, informed personnel [4]. As a consequence, a medical network system is considered as a network requiring high security that excellent protections and managerial strategies are inevitable to prevent illegal events and external attacks from happening. Since the debut of this era, one of the most intelligible terms of medical information technology and communication is the security of medical records and patient’s personal information like unique id, name of the patients, disease name, etc. Steganography is a method of secret communication in which secret information is embedded into other information, various multimedia files.

However, in case of cryptography the secret information is encrypted by a key and an algorithm and sent through the transmission channel. Cryptography and steganography are used for providing security to the transmitted medical records and patient’s personal information over the internet and networking. The advantage of steganography over cryptography is that it keeps the presence of data obscure and secret. The proposed method provides two levels of security. Firstly, the medical records and patient’s personal information are encrypted using RSA public key cryptographic algorithm and secondly the encrypted data are then concealed into the LSB plane of different Brain disease cover images using the diagonal queue substitutions, thus the strength of steganography can be increased with cryptography. Hiding the data into least significant bits of cover image does not much affect its visual appearance quality.

There are numerous procedures used to hide a variety of multimedia secrets inside distinguished multimedia files. Anderson and Petitcolas discussed some limitations in steganography methods. They proposed an information-theoretic method using Shannon’s theory for perfect security of data [5]. Mohammad and Jantan suggested the LSB (Least Significant Bit) procedure in which one bit of secret data is substituted at the 8th LSB position of every byte of the coated file, if the entropy and correlation values of stego image and the cover image show equality after enciphering, then it represents that the process is safe [6]. Srivastava and Mathur have proposed the effect of applying well-known steganography techniques on various statistical models of natural images. On one side, they retrieved that some popular stego-algorithms consistently bias these statistics for some of the most basic models. On the other side, the intrinsic variability of these statistics is so large, for the class of images discussed, that this bias induced by hiding “unnatural” information is not enough, in general, to transmit the results outside of the “natural” range unless the knowledge of the implanting algorithm is available and exploited [7]. Juneja and Sandhu [8] described a new LSB array-based mechanism. It combines whole LSB bits of diverse pixels as an LSB array. The encrypted message block has been mapped to LSB array. A matching process has been applied to find out the maximum matching portion for embedding secret data. Swain and Lenka [9] proposed a new steganography technique based on LSB array. The image is transformed to binary form. Four LSB arrays such as LSB0, LSB1, LSB2, and LSB3 are used. One of the four arrays is obtained and formed on the basis of the length of the secret message. For large size messages, LSB3 array is used. The different words of the secret data are mapped on the chosen array, where maximum match is found, which obscured the data and start indices are noted down. Once a word is embedded in a particular region of the array that region is made unavailable for another word. The RSA algorithm is used to encrypt the length of each word and their start indices altogether. The cipher text is compressed and then embedded at some reserved area in the image. This reserved area was not used in forming the used LSB array. In another method, proposed by Swain and Lenka, a steganography methodology using four LSB arrays is suggested. LSB, LSB1, LSB2, and LSB3 arrays are constructed. Different blocks are mapped to different LSB array and embedding will be performed at a maximum matching region of the corresponding LSB array. By this mechanism, the security and capacity have been improved [10]. Pixel value differencing method has been proposed by Wu and Tsai [11]. A cover image is used for secret data substitution using the difference values of the two-pixel blocks. A two-way block matching procedure and the hop embedding scheme is suggested to hide a secret image data inside a cover image [12]. Kumar and Roopa proposed the same block matching scheme and the hop embedding technique. They improved tamper proofing, so that any attack cannot modify the content of the embedded data in the cover image [13]. Parvez and Gutub described a steganography methodology using RGB intensity values of the pixel [14]. They suggest the concept of an indicator channel and remaining two channels to hide secret data bits. The last two bits of the indicator channel will give information about the hiding data in the other two channels. Zhang and Wang [15] found that pixel value differencing steganography is vulnerable to histogram-based attacks and suggested a modified version to enhance security. Chang and Tseng [16] proposed a new concept based on various sided side matching techniques. The two-, three-, and four-sided side matching methods use the side information of the various side neighboring pixels to take decision for data hiding. Nag et al. suggested a novel method in a steganographic system based on the affine encryption algorithm and embed the secret data at the LSB position in order to advise a solid security and imperceptible visual quality to secret data [17]. Three steganography techniques are described by Maiti et al. for hiding secrets in coated image. They have used last two least significant bits for embedding secrets in diagonal pixels of the cover image. Public key cryptography is used to encrypt the secret data asymmetrically [18]. Thiyagarajan and Aghila [19] proposed a new steganography methodology for hiding patient information inside a brain cover image using a dynamic key produced by graph 3 coloring problem. This technique ensures reversibility of original Brain image after extracting the embedded secret data from the Brain stego image.

Our novel approach can be understood by referring the following divisions. In division 2, the proposed methodology is discussed, in division 3, the architecture of the proposed algorithm is discussed, in division 4, work methodology is suggested, in division 5, sender side methodology is introduced, in division 6, receiver side methodology is discussed, in division 7, results and discussion are presented, and finally the work is concluded.

## Proposed methodology

A variety of public key cryptography and data structure methods exist with which most of us are familiar.

### Rabin cryptosystem

The Rabin cryptosystem is a public key enciphering technique [20]. It is established on number-theoretic problems allied to the stiffness of integer factoring and computing square roots modulo of composite number, which is straightforward when the factorization is familiar, but very complex when it is concealed. The Rabin cryptosystem requires a receiver’s public key to encrypt the text and a private key to decrypt it.

The first step is to select the key which is defined by

1 $$ K \, = \, \left\{ {n, \, p, \, q} \right\}, $$

where *p* and *q* are, respectively, prime numbers and private key such that

2 $$ p, \, q \equiv 3 {\text{ mod 4}} . $$

The receiver’s public key is

3 $$ n \, = \, pq. $$

Then, to encipher the message m, the encryption function is applied:

4 $$ {\text{e}}K\left( m \right) \, = \, m^{2} \bmod \, n \, = \, c. $$

The ciphertext, c, is the output.

Now, the encrypted message can be sent to the receiver. When the message extends the destination, the decryption function is applied:

5 $$ dK\left( c \right) \, = \, \sqrt { \, c \, \bmod \, n} . $$

Since the enciphering function *eK* is not an injection procedure, the decryption is not ambiguous. There are four square roots of c mod n (*c* *=* *m*^*2*^ mod *n*), so there are four possible messages, *m*.

The decryption tries to determine m such that

6 $$ m^{2} \equiv c \, \bmod \, n. $$

And this is equivalent to solving the two congruences:

7 $$ z^{2} = \, c \, \bmod \, p, $$

8 $$ z^{2} = \, c \, \bmod \, q. $$

Then

9 $$ m_{p} = \, c^{p + 1/4} \bmod \, p, $$

10 $$ m_{q} = \, c^{q + 1/4} \bmod \, q. $$

Finally, the four square roots of c mod n can be generated by introducing the Chinese remainder theorem to the system of congruences:

11 $$ + m_{p} \bmod \, p, $$

12 $$ - m_{p} \bmod \, p, $$

13 $$ + m_{q} \bmod \, q, $$

14 $$ - m_{q} \bmod \, q. $$

### Security of the Rabin cryptosystem

The security of Rabin cryptosystem is based on its decryption function, since the decryption function of this cryptosystem is based on computing square roots modulo of composite number *N*. It is feasible to demonstrate that the exploiting of cryptanalysis the Rabin cryptosystem is equivalent to the exploiting of factoring of a composite number. The Rabin cryptosystem is secure against chosen-plaintext attacks; however, the system can be broken using ciphertext attacks enabling the attacker to know the private key.

### Queue

**Fig. 1:**
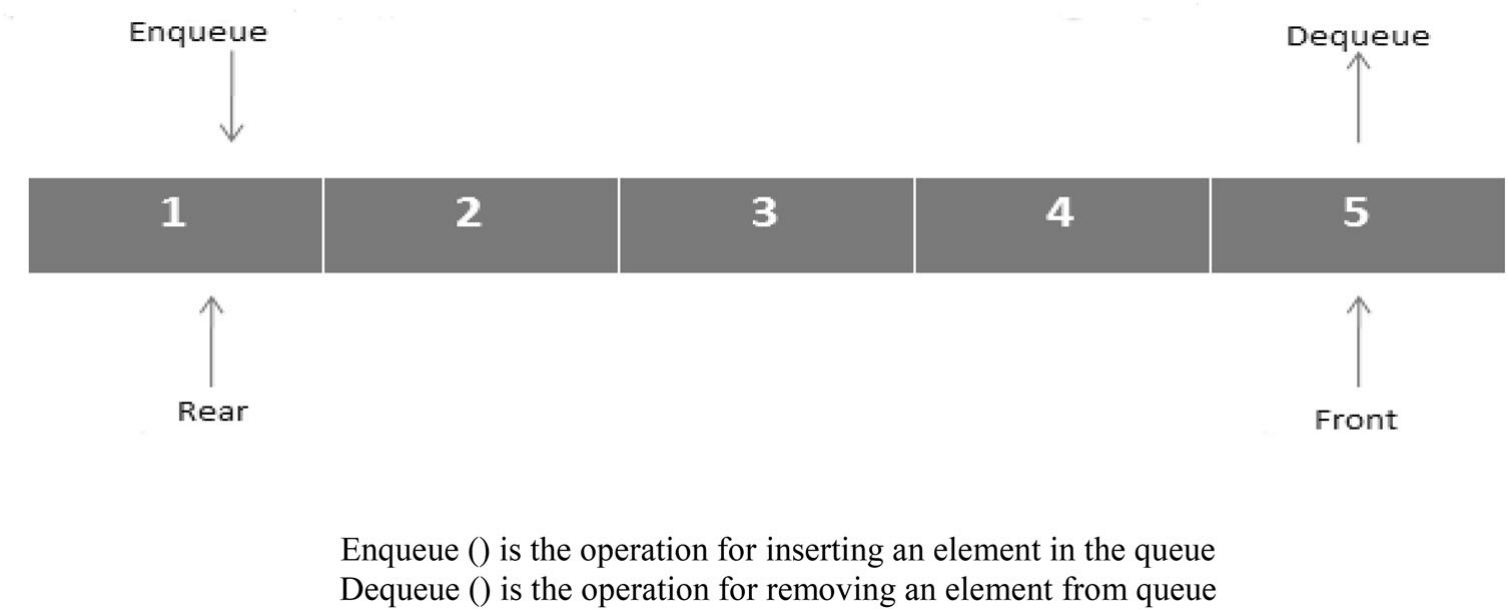
Queue representation

The linear data structure or abstractly a sequential collection is called a queue [21]. The principal operations on the collection of entities are the addition of them to the rear terminal position, known as enqueuing, and deletion of entities from the front terminal position, known as dequeuing. This makes the queue follow a First-In-First-Out (FIFO) property of data structure. The first incoming data to the queue will remove first from the queue as shown in Fig. 1.

## Architecture of the proposed method

**Fig. 2:**
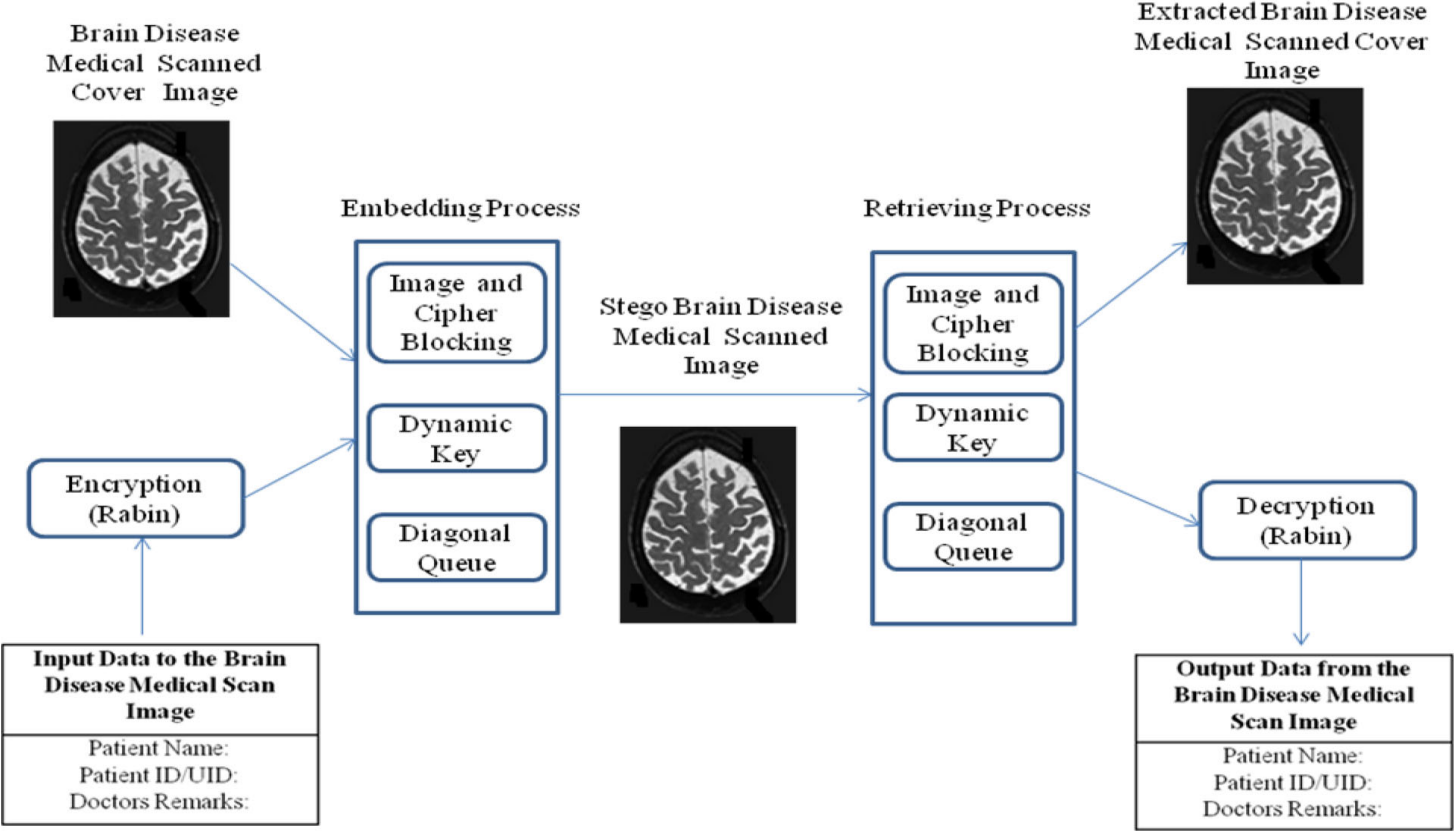
Architecture for medical brain image steganography using diagonal queue

The proposed architecture is shown in Fig. 2. It is mainly focused on providing a solution for transferring and sharing medical record and personal identification information of patient without any compromise in security. All the reputed medical centers while sending medical documents over the internet always use encryption to authenticate the medical information as well as protect leakage of information about their centers from rivals’ or intruders’ misuse. We have proposed an architecture using a secure crypto–stegano algorithm which is far more secure than many systems being used for the purpose of secretly sending the medical data. This architecture consists of Rabin public key cryptosystem to enhance the secrecy and confidentiality of the medical data by converting medical record and personal identification information of patients into cipher text. In embedding process, Brain disease cover image is divided into various blocks and cipher text also divided into a number of blocks as well as into sub-blocks. The random selection of blocks and sub-blocks will be done by a dynamic key. Now the each Brain disease cover image block is organized in various diagonal queues. Only some diagonal queues are used for secret cipher embedding. Diagonal queues for embedding secret cipher data are selected robustly at various bit locations among the 8th to 5th bit LSB sequentially. In retrieving process, the reverse mechanism is followed to get patient medical information and Brain disease medical cover image.

## Work flow diagram

**Fig. 3:**
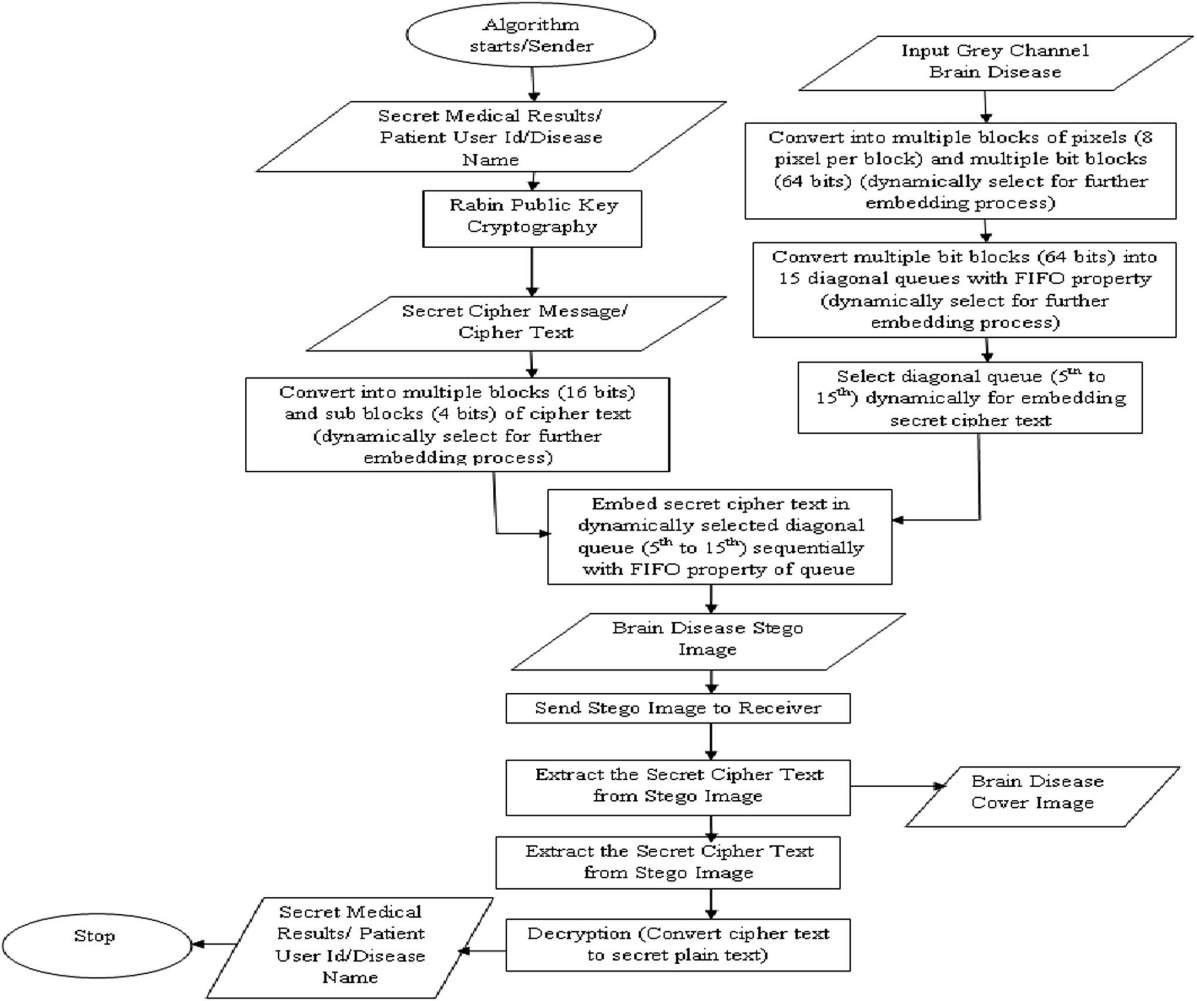
Work flow diagram of the proposed methodology

It is a challenging process for us to combine the two technologies: Rabin public key encryption algorithm and diagonal queue LSB substitution. Figure 3 shows the work flow of the proposed algorithm.

## Sender’s side methodology

### Cover image and secret message

In our novel opted system, first of all we take a grayscale scanned image of Brain disease as a cover image and a secret message which may be Brain disease medical record and patient’s personal information like unique id, name of the patient, and disease name to be embedded in the Brain disease cover image of patient. Brain disease cover image will keep some reserved bytes (say, 4045 bytes) at the beginning of the image for embedding the number of secret cipher message blocks and sub-blocks, dynamic key value, secret cipher message block length, number of diagonal queues in one cover image block, number of cover image blocks, and the number of diagonal queues for data embedding (i.e., 5th to 15th).

### Rabin cryptosystem

This novel approach of image steganography uses Rabin encryption technique to encrypt the secret medical data (Brain patient data) before embedding. Encryption includes medical record and patient’s personal information like unique id, name of the patient, and disease name for converting it into the cipher text. The Rabin cryptosystem requires a public key to encrypt the secret medical (Brain disease) data of patient and a private key (prime numbers) to decrypt it. Now, to select the suitable values for private keys, we explain one example here. Suppose we input the secret message 3 and two integer values. This is not only the input for Rabin cryptosystem, but also to the complete system. Rabin cryptosystem is just one part of the complete system.

Here the conversion process to get decimal values is shown below; this will give us the input for Rabin cryptosystem:

Convert “3” and “2” into ASCII decimal values → 51 and 50.

Convert both of these into binary → 110011 and 110010.

Now, pad the message with itself → 110011110011 and 110010110010.

Convert this back to decimals → 3315 and 3250.

The above 2 decimal numbers are now input to Rabin cryptosystem. Here, the input secret message is 3 and 2, but after conversion we have a very large value.

Also, for Rabin cryptosystem, 0 ≤ message ≤ *n* − 1 where *n* = p*q and *p*, *q* are prime numbers.

So if we take *p*, *q* < 131, our condition will not meet. Hence, we will get wrong answers.

So it is mandatory to work with *p*, *q* ≥ 131

So, if we enter any input in GUI (Graphical User Interface), we have to enter *p*, *q* ≥ 131 to get plain text value back.

After decryption, Rabin cryptosystem gives four values of medical data plain text corresponding to one cipher text. So only authorized receiver can identify the correct medical record.

At the end of this process, we obtain secret cipher texts enciphered from the original secret medical data (Brain disease data) of patient to be inserted in the Brain disease cover image.

### Diagonal queue

After Rabin encryption, cipher text will be obtained. Now cipher text will be divided into blocks and each block has 16 bits. After that, each block is divided into equally distributed 4-bit sub-blocks. Now, the Brain disease cover image will be divided except some reserved location, i.e., byte numbers 3045 to 4045, into a number of image blocks and each image block has 8 pixels with 64 bits. Then the each image block’s 64 bits are organized in 15 diagonal queues from right bit to left bit insertion using FIFO property of queue from top to bottom.

### Diagonal queue embedding

In this process, diagonal queue insertion technique is used for embedding the secret cipher values in the various Brain disease cover images. Now, embedding the secret cipher text in the Brain disease cover image using a diagonal queue is done as follows:

- Embed the number of secret cipher message blocks and sub-blocks, dynamic key value, secret cipher message block length, number of diagonal queues in one Brain disease cover image block, number of Brain disease cover image blocks, and the number of diagonal queues for data embedding (i.e., 5th to 15th) in some reserved location, i.e., byte numbers 3045 to 4045.
- Convert the values of cipher text and Brain disease cover image into binary form.
- Now, dynamically select one block and its sub-blocks of secret cipher message and assign them to one block of the Brain disease cover image, which are represented by diagonal queues for embedding.
- Embedding will be done in dynamically selected diagonal queues (i.e., 5th to 15th) sequentially using FIFO property of queue from right to left since we can embed only 8th to 5th bit LSB position for better visual quality of Brain disease stego image.
- Go to step (c).

This process is continued until all the cipher data blocks are not empty and all the secret cipher text is not embedded in diagonal queues sequentially and the resultant Brain disease stego image is sent to the receiver.

An example of the embedding procedure is shown in Table 1:

**Table 1:** (a), (b), (c), (d), and (e) are cipher text blocks, cipher text sub-blocks, Brain disease cover image blocks, cover image block into bits, and diagonal queues respectively

1. Suppose we have the following data
a. *N* blocks of cipher text: 1 × 16
(a) Cipher text blocks
b. 4 sub-blocks of each N block of cipher text: 1 × 4
(b) Cipher text sub-blocks
c. *M* block of 8 pixels each, from Brain disease cover image: 1 × 8
(c) Brain disease cover image blocks
d. *M* block of 64 bits each. We can obtain this by converting the above block into bits: 8 × 8
(d) Cover image block into bits
2. From the above matrix, we have 15 diagonal queues from right to left inserted bits, and among the 5th–15th diagonal queues are eligible for secret cipher data embedding
(e) Diagonal queues
3. The above-shown bold bits are the diagonal queue LSB bits (i.e., 8th to 5th), which can be swapped with the cipher text bits using FIFO property of queue
4. Now, we will select one of these eligible diagonal queues, dynamically
5. We will also select one block from the *N* blocks and sub-blocks dynamically
6. We will then put the selected ciphertext bits in selected diagonal queue at 8th to 5th bit LSB position sequentially

## Receiver’s side methodology

Now, the retrieval of the secret cipher text and medical data (Brain patient data) plain text from the Brain disease cover image is done as follows:

- Retrieve the number of secret cipher message blocks and sub-blocks, dynamic key value, secret cipher message block length, number of diagonal queues in one Brain disease cover image block, number of Brain disease cover image blocks, and the number of diagonal queues for data embedding (i.e., 5th to 15th) from some reserved location, i.e., byte numbers 3045 to 4045.
- Retrieval will be done from each Brain disease cover image pixel block from dynamically selected diagonal queues (i.e., 5th to 15th) sequentially using FIFO property of queue from right to left side, since embedding has been performed only at the 5th to 8th LSB position for better visual quality of Brain disease stego image.
- Go to step (b) until all the secret cipher text is retrieved.
- Now, take all the cipher bits and arrange them in sub-blocks and blocks according to their dynamic key values.
- Convert the binary value into integers.
- Now, apply Rabin decryption to secret cipher message to get medical data plain text back. After decryption, Rabin cryptosystem gives four values of medical data plain text corresponding to one cipher text. So only authorized receiver can get the correct brain medical information with its private key.
- Stop.

## Results and discussion

**Fig. 4:**
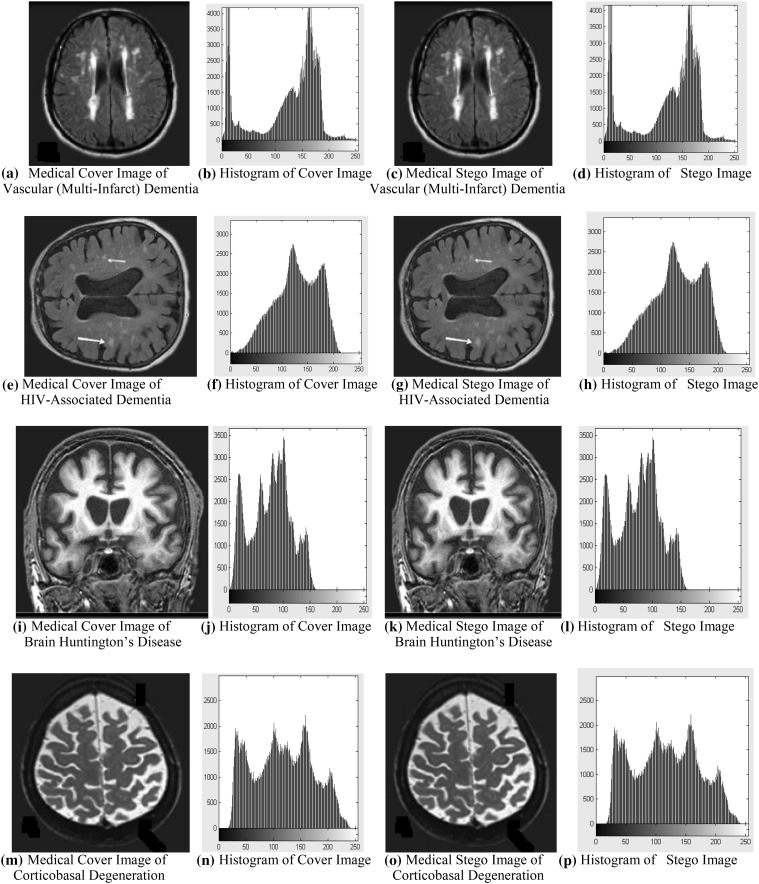
**a**, **e**, **i**, **m** Brain disease medical cover images and **c**, **g**, **k**, **o** their stego images; **b**, **f**, **j**, **n** histograms of Brain disease medical cover images and **d**, **h**, **l**, **p** their stego images

The simulation and experimentation have been done using MATLAB for Rabin cryptosystem with diagonal queue steganography. Graphical user interface is used to implement this to make convenient for the user to handle it. The resultant simulated outcome for different Brain disease cover images and their stego images are displayed in Fig. 4. If the histograms are also considered, then there is a negligible amount of difference between the histogram of the various original Brain disease cover images and their stego images. Histograms for various original images and their stego images are also shown in Fig. 4.

The patient information used in this work is shown in Table 2.

**Table 2:** Medical Record of the Patient

Input data to the Brain disease medical scan cover image	Output using the proposed methodology
Patient name: XXX	Patient name: XXX
Patient ID/UID: XX	Patient ID/UID: XX
Doctors remarks: X	Doctors remarks: X

The clause peak signal-to-noise ratio is a technical terminology that defines the ratio between the maximum power of a signal and the power of damaged noise. The representation/quality of the signal is affected due to corrupted noise. An important index to readjust the quality of reformation of steganographic images is the peak signal-to-noise ratio. The original cover image acts like a signal, and the noise is the defect included by some steganography mechanism. The PSNR, MSE, and maximum embedding volume at divergent medical payloads for different Brain disease cover images of various sizes are given in Table 3. PSNR is calculated in decibels (dB). A high-quality stego image should aspire for 40 dB and above [4].

**Table 3:** Observed Capacity, MSE, and PSNR value (different cover images of same/different sizes with various secret cipher data of same/different sizes)

Brain disease cover image (*.bmp)	Brain disease cover image size (in kilobytes)	Quantity of cipher embedded (in bytes)	Maximum embedding volume (in kilo Bytes)	Percentage of embedding volume w.r.t image size (%)	MSE	PSNR (in dB)
Vascular (Multi-Infarct) Dementia	262	256	89.32	34	0.0021	74.77
Vascular (Multi-Infarct) Dementia	262	1024	89.32	34	0.0054	70.37
HIV-Associated Dementia	262	256	86.67	33	0.0026	73.56
HIV-Associated Dementia	262	1024	86.67	33	0.0056	70.68
Brain Huntington’s Disease	262	256	84.49	32	0.0049	73.02
Brain Huntington’s Disease	262	1024	84.49	32	0.0038	71.08
Corticobasal Degeneration	262	256	85.81	33	0.0022	75.46
Corticobasal Degeneration	262	1024	85.81	33	0.0041	71.57
Vascular (Multi-Infarct) Dementia	1048	256	387.20	36	0.0003	83.46
Vascular (Multi-Infarct) Dementia	1048	1024	387.20	36	0.0010	78.39
HIV-Associated Dementia	1048	256	383.49	37	0.0004	82.18
HIV-Associated Dementia	1048	1024	383.49	37	0.0011	77.29
Brain Huntington’s Disease	1048	256	379.14	36	0.0004	82.79
Brain Huntington’s Disease	1048	1024	379.14	36	0.0010	78.69
Corticobasal Degeneration	1048	256	380.39	36	0.0003	83.09
Corticobasal Degeneration	1048	1024	380.39	36	0.0010	77.76

PSNR outcome is defined by the mean square error (MSE) for two P × Q monochrome images, where x and y are image coordinates, SG_xy_ (stego image) and CV_xy_ (cover image), and one of the images is approved a noisy surmise of the other:

15 $$ {\text{MSE}}\; = \;\frac{1}{PQ}\;\mathop \sum \limits_{x = 1}^{P} \mathop \sum \limits_{y = 1}^{Q} \;\left( {{\text{SG}}_{xy} - {\text{ CV}}_{xy} } \right), $$

16 $$ {\text{PSNR}} = { 1}0{ \log }_{ 10} \left\{ {{\text{CV}}_{ \hbox{max} }^{ 2} /{\text{MSE}}} \right\}, $$

where CV_max_ is the maximum 255-pixel value, for 8-bit cover images [4].

In this paper, the results are measured by giving multiple levels of security to the secret Brain medical data. In the very beginning phase, encrypt the secret medical record and patient’s personal information using Rabin public key cryptosystem and later embedding secret cipher information in dynamically selected diagonal queues (i.e., 5th to 15th) sequentially using FIFO property of queue from right to left side, since we are embedding only the 8th to 5th bit LSB position for better visual quality of Brain disease stego image.

From Table 3, the results are analyzed. If Brain disease cover images such as Vascular (Multi-Infarct) Dementia, HIV-Associated Dementia, Brain Huntington’s Disease, and Corticobasal Degeneration are of 262 kilobytes size and secret data size is 256 bytes, then PSNR and MSE value will be in the range from 73.56 to 75.46 dB and 0.0049 to 0.0022, respectively, and if data size increases to 1024 bytes, then PSNR and MSE value will be in the range from 70.37 to 71.57 dB and 0.0054 to 0.0041, respectively. If Brain disease cover images’ size increases to 1048 kilobytes and secret data size is 256 bytes, then PSNR and MSE value will be in the range from 82.18 to 83.46 dB and 0.0004 to 0.0003, respectively, and if secret Brain medical data size increases to 1024 bytes, then PSNR and MSE value will be in the range from 77.29 to 78.69 and 0.0011 to 0.0010, respectively. In Vascular (Multi-Infarct) Dementia Brain image, maximum embedding capacity is 89.32 and 387.20 kilo bytes which are 34 and 36 %, respectively, of the Brain image size. In HIV-Associated Dementia Brain image, it is 86.67 and 383.49 kilo bytes, which is 33 and 37 %, respectively, of the Brain image size. In Brain Huntington’s Disease image, it is 84.49 and 379.14 kilo bytes, which is 32 and 36 %, respectively, of the Brain image size. In Corticobasal Degeneration image, it is 85.81 and 380.39 kilo bytes, which is 33 and 36 %, respectively, of the Brain image size. So by result analysis it can be noticed that by increasing the Brain disease cover image size and decreasing the secret brain medical data size, PSNR value will be increased up to 83.46 dB and MSE value will be decreased up to 0.0003 as well as maximum embedding capacity is increased up to 37 %. So that performance will be high with respect to PSNR, MSE, and maximum embedding capacity value. From Fig. 4, one can observe that there are no visual artifacts with the stego images and histograms, and it looks exactly the same as the corresponding original Brain disease cover images.

Steganographic methods’ performance can be observed by the three valuable specifications: secrecy, volume/capacity, and visual imperceptibility [4]. Secrecy is used to protect data from unauthenticated attackers or intruders. The hiding capacity should be enough to obscure the data in a cover image. Visual quality of stego image should be such that no one can claim about imperceptibility [22].

Our image steganography approach is exceedingly secure for sending medical record and personal information of patients, since it uses allocation of message blocks to image blocks through dynamic selection and embedding of secret cipher information will be performed in dynamically selected diagonal queues (i.e., 5th to 15th) sequentially using FIFO property of queue from right to left, since we are embedding only at 8th to 5th bit LSB position for better visual quality of Brain disease stego image. The existing intruders and attacks cannot identify the existence of steganography. Moreover, we are hiding the cipher text, not the medical data directly, which increases one level of security. The enciphering algorithm is the Rabin public key cryptographic algorithm.

Using Table 4, the comparison of the proposed scheme is shown on the basis of minimum calculated PSNR, embedding capacity, and visual imperceptibility with the different schemes proposed by other researchers in this field. Compared to other algorithms, our algorithm is stronger and can be used for securing different varieties of secret Brain medical data.

**Table 4:** Comparison with other Researchers

Research article	Minimum calculated PSNR(dB)	Capacity	Visual imperceptibility
Thiyagarajan and Aghila [19]	65.53	Good	Better
Swain and Lenka [10]	50.50	Medium	Better
Wang and Chen [12]	44.20	Medium	Better
Kumar and Roopa [13]	44.15	Medium	Better
Wu and Tsai [11]	37.90	Low	Average
Zhang and Wang [15]	36.00	Low	Average
Chang and Tseng [16]	33.53	Low	Average
Nag et al. [17]	30.48	Very low	Not good
Proposed algorithm	70.37	Very good	Best

## Conclusion

In this paper, a novel secret transmission scheme has been proposed using the notion of opacity with respect to a diagonal queue least significant bit substitution, which is an extremely effective alternative for transmitting secure medical records and patient’s personal identification information along with the appropriate medical Brain disease image. The secret message blocks and sub-blocks are allocated dynamically by the sender to the Brain disease cover image blocks with respect to diagonal queues, which increases security levels and gives dynamic effect to the proposed algorithm. The proposed algorithm has used Rabin public key cryptosystem at cryptography level to provide confidentiality of Brain medical information of patient at medical data center and end-to-end communication, since it is computationally secure associated chosen-plaintext attack, tremendously smaller susceptible to occurrence investigation attack and enciphered message attacks. It also shows the difficulty of integer factoring. At steganography level, least significant bit substitutions using diagonal queues have been used to protect sensitive medical information of patient like HIV report and baby girl fetus from leakage in transmission channel when resources are shared among multiple transmission holders. Using the multilevel encoding approach presented, the medical Brain disease image itself may be hidden inside. From the results and histogram analysis, it is concluded that PSNR, MSE values, and the percentage of maximum embedding capacity are better as compared to some of the existing algorithms and imperceptibility distortion cannot be measured from the corresponding medical Brain disease stego images.
